# Alleviation of Methamphetamine Sensitization by Partially Lesioning Dopaminergic Terminals with 6-Hydroxydopamine in Nucleus Accumbens

**DOI:** 10.1177/09636897211052300

**Published:** 2021-11-08

**Authors:** Shu-Chun Chen, Hsi Chen, Seong-Jin Yu, Yun-Hsiang Chen, Yun Wang

**Affiliations:** 1 Center for Neuropsychiatric Research, National Health Research Institutes, Miaoli, Taiwan; 2 Graduate Institute of Applied Science and Engineering, Fu-Jen Catholic University, New Taipei City, Taiwan; 3 Department of Life Science, Fu-Jen Catholic University, New Taipei City, Taiwan

**Keywords:** methamphetamine, addiction, nucleus accumbens, 6-hydroxydopamine

## Abstract

Amphetamine-type stimulants have become important and popular abused drugs worldwide. Methamphetamine (Meth) sensitization, characterized by a progressive increase in behavioral responses after repeated administration, has been reported in rodents and patients. This behavioral effect has been used as a laboratory model to study drug addiction and schizophrenia. The mesolimbic dopaminergic pathway plays a significant role in the development of Meth behavioral sensitization. Previous studies have reported that the ablation of nucleus accumbens (NAc) by electrolytic or thermal lesioning attenuates addictive behavior to opioids in animals. However, these studies were only conducted in opioid addictive rodents. Furthermore, these ablation procedures also damaged the non-dopaminergic neurons and fibers passing through the NAc. The purpose of this study was to examine the therapeutic effect of NAc lesioning by a selective dopaminergic toxin in Meth-sensitized animals. Adult mice received repeated administration of Meth for 7 days. Open-field locomotor activity and stereotype behavior were significantly increased after Meth treatment, suggesting behavior sensitization. A partial lesion of dopaminergic terminals was made through stereotaxic administration of dopaminergic toxin 6-hydroxydopamine (6-OHDA) to the NAc in the Meth -sensitized mice. Meth behavioral sensitization was significantly antagonized after the lesioning. Brain tissue was collected for qRT-PCR analysis. Repeated administration of Meth increased the expression of tyrosine hydroxylase (TH), BDNF, and Shati, a marker for Meth sensitization, in the NAc. Treatment with 6-OHDA significantly antagonized the upregulation of TH and Shati. Taken together, these data suggest that local administration of 6-OHDA mitigated Meth sensitization in chronic Meth-treated animals. Our data support a new surgical treatment strategy for Meth abuse.

## Introduction

Amphetamine-type stimulants (ATS) have become important and popular abused drugs worldwide. According to a report from the United Nations Office on Drugs and Crime, an estimated 34.2 million people had used amphetamines, and half of the global estimated amphetamine users reside in Asia^1^. In addition, an epidemiology study also indicates the high or rising prevalence of methamphetamine (Meth) use in China^2^ and Northern America^3,4^.

The current treatment of Meth abuse has mainly relied on psychosocial intervention, such as cognitive behavior therapy, contingency management, motivation therapy, counseling. More than 100 pharmacological therapies have been clinically examined over the past 30 years. None of these drugs has significant and reproducible benefits. No medication has been approved in any country with the indication to treat ATS disorders ^1^. Unlike methadone for treating opioid addiction, there is no replacement or substitutional therapy for Meth abuse.

Meth sensitization, characterized by a progressive increase in behavioral responses after repeated administration, has been reported in rodents^5^ and patients^6^. This behavioral effect plays a key role in Meth-mediated plasticity^7,8^ and has been used as a laboratory model to study drug addiction^9^ and schizophrenia^10^. In rodents, repeated Meth exposure induced long-lasting sensitization. The sustained hyperactive response lasts from >7 days^11,12^ to several months^13^. The mesolimbic dopaminergic pathway plays a significant role in developing Meth behavioral sensitization^14,15^. Meth sensitization is associated with increases in extracellular dopamine (DA) levels in the nucleus accumbens (NAc) and striatum^16,17^, upregulation of BDNF^18^ and Shati, a potential marker for Meth sensitization^19,20,21^, in the NAc.

Repeated Meth use can lead to persistent structural changes in the reward pathway of the addictive brain^22^. Chronic use of ATS increases dendritic spines of dopaminergic neurons^23^. Repeated exposure to amphetamine alters the number of dendrites and the density of dendritic spines of neurons, and the spine density of medium spiny neurons in the NAc^24,25^. A similar response was also found in rat PC12 cells that repeated administration of amphetamine induces neurite outgrowth and enhances amphetamine–stimulated dopamine release^26,27^. Other ultrastructural modifications include the changes in dopamine D2 or D3, mu-opioid, or glutamate receptors in striatum and NAc^28–31^. These structural changes in the mesolimbic system limit pharmacological therapy for Meth addiction and behavioral sensitization.

A few studies have been conducted to control opioid-mediated persistent structural changes in the reward pathway and addictive behavior by surgery. Electrolytic lesioning the NAc reduced conditioned place preference in morphine-addicted rats^32^. In another study, lesions were introduced by passing a direct current (DC, 0.5 mA, 0.5 s width, 10-70 s) to the NAc or ventral tegmental area. A significant reduction of drug priming relapse was found after 9-day abstinence in chronic morphine-treated rats (4 mg/kg × 10 d)^33^. However, there are still a few unsolved questions. For example, these studies were only conducted in opioid addictive rodents. It is not clear if the ablation of NAc alters addictive behavior for Meth. Furthermore, these ablation procedures also damage the non-dopaminergic neurons and fibers passing through the NAc.

The purpose of this study was to examine the potential therapeutic effect of lesioning dopaminergic terminals against Meth sensitization. We found that administration of 6-hydroxydopamine 6-OHDA, a dopaminergic neurotoxin, partially lesioned the DA terminals in NAc, normalized the behavior sensitization, and significantly antagonized the upregulation of TH and Shati in NAc. Our data suggest that local administration of low dose 6-OHDA mitigated Meth sensitization in chronic Meth-treated animals. Our data support a new surgical treatment strategy for Meth abuse.

## Materials and Methods

### Animals

Adult male CD-1 mice were purchased from the Lasco, Taiwan. Experimental procedures followed the guidelines of the “Principles of Laboratory Care” (National Institutes of Health publication no. 86-23, 1996). Animals were housed in a 12-hr dark (7 pm to 7 am) and 12-hr light (7 am to 7 pm) cycle.

### Meth-Mediated Behavioral Sensitization

Mice were treated with Meth (2.5 mg/kg/day, s.c.) or saline for 7 days. On the day of behavior test, animals were first placed into locomotor chambers (Accuscan, Columbus, Ohio, USA) for 1-hour acclimatization. Locomotor activity was recorded for 1-hour after injection of Meth (1 mg/kg, s.c.). Analysis was conducted using the total distance traveled (TOTDIST; the distance traveled in centimeters), horizontal activity (HACTV; the total number of beam interruptions that occurred in the horizontal sensors), movement time (MOVTIME, the amount of time in ambulation), and stereotypic movement number (STRCNT).

### 6-OHDA Lesioning

Mice were anesthetized with chloral hydrate (400 mg/kg, i.p.) and placed in a stereotaxic frame. 6-OHDA HBr (2.76 µg/µl × 1 µl in 0.9% NaCl containing 0.2 mg/ml ascorbic acid) was injected into the bilateral NAc over 4 min through a 10-µl Hamilton microsyringe. The tip of microsyringe was moved to the desired target locus (coordinates: A/P +1.3 mm, M/L 1.0 mm, D/V 3.4 mm, according to Paxinos and Franklin’s “the Mouse Brain”) using micromanipulators attached to the stereotaxic frame. The speed of injection (0.33 µl/min) was controlled by a syringe pump (Micro 4, WPI, Sarasota, FL). The needle was removed 5 min after each injection. A piece of bone wax was placed on the burr hole to prevent the leakage of fluid. The wound was sutured or clipped. Body temperature was monitored with a thermistor probe and maintained at 37°C with a heating pad during anesthesia. After recovery from the anesthesia, animals were housed in their home cages

### Quantitative Reverse Transcription PCR (qRT-PCR)

Brain NAc tissues were collected for qRT-PCR analysis. Total RNAs were isolated using TRIZOL Reagents (Life Technologies, #15596-026), and cDNAs were synthesized from 1 µg total RNA using a RevertAid First Strand cDNA Synthesis Kit (Thermo Scientific, #K1622). The primers for qRTPCR are shown in Table 1. qRT-PCR was carried out using Luminaris Color HiGreen qPCR Master Mix, low ROX (Thermo Scientific, #K0371), and Applied Biosystems QuantStudio 3 Real-Time PCR System. Expression and normalization of the target genes (Shati, TH, and BDNF) were calculated relative to the endogenous reference gene (GAPDH) with a modified delta-delta-Ct algorithm that takes specific gene-specific amplification efficiency into account for accurate calculation. All experiments were duplicated.

**Table 1.** Oligonucleotide Primers Used for Quantitative RT-PCR.

**Gene**	**Direction**	**Sequence (5′→3′)**	**Reference**
Shati	Forward	GGGTGGCCGGGTAGGTGGAA	^40^
	Reverse	GGCAGTGCCCAGCCCTTCCT	
TH	Forward	GTCTCAGAGCAGGATACCAAGC	^41^
	Reverse	CTCTCCTCGAATACCACAGCC	
BDNF	Forward	CAGAGCAGCTGCCTTGATGTT	^42^
	Reverse	GCCTTGTCCGTGGACGTTTA	
GAPDH	Forward Reverse	CATCACTGCCACCCAGAAGACTG ATGCCAGTGAGCTTCCCGTTCAG	OriGene Technologies Inc. CAT#: MP205604

### Statistics

Data are presented as mean ± s.e.m. Unpaired Student’s *t*-test, 1- or 2-way ANOVA were used for statistical comparisons, with a significance level of *P* < 0.05. In the event of multiple comparisons, a posthoc Newman-Keuls test (NK test) was performed. All analyses were performed with Sigmaplot ver. 12.5 software (Systat Software Inc., Chicago, IL, USA).

## Results

### Meth-mediated Behavioral Sensitization

**Figure 1.**
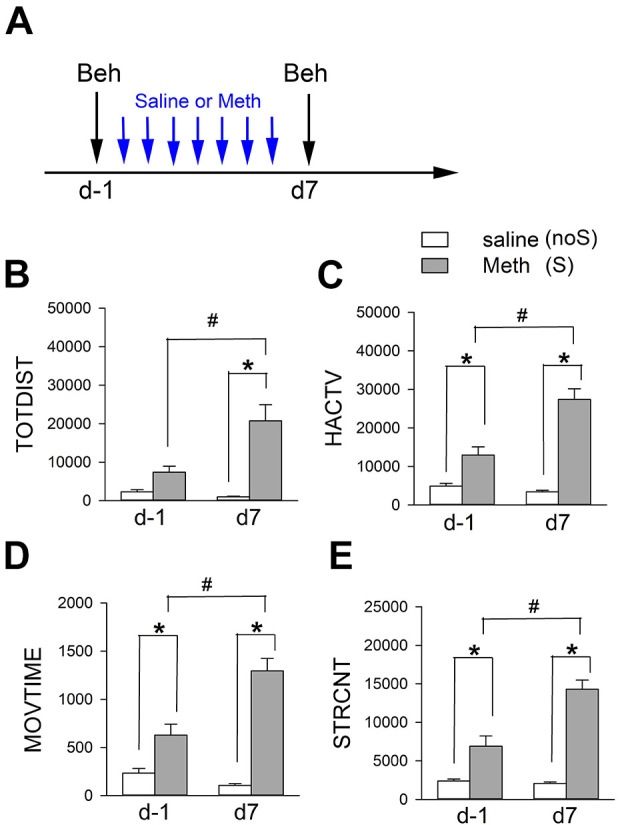
Chronic treatment with Meth induces behavioral sensitization. (A) Timeline (induction of Meth sensitization). Animals were separated into two groups. Mice in the sensitized group (S) were stimulated with a daily dose of Meth (2.5 mg/kg/d, from day 0 to day 6) while the control animals (noS) received daily saline (day 0 to day 6). (B–E) The locomotor activity was recorded for one hour after injection of low dose of Meth (1 mg/kg, S group) or saline (noS group) on days -1 and 7. Meth significantly increased locomotor and stereotype behaviors than saline control on day -1 and day 7 (S vs. noS). Meth-induced hyperactivity was further enhanced after repeated injection of Meth on day 7 (#*P* < 0.05, day 7 vs. day -1; *P < 0.05, Meth vs. saline, two-way ANOVA+NK test).

Animals were separated into 2 groups (Meth -sensitized or S group and non-sensitized or noS group) to receive chronic Meth or saline. Meth sensitization was induced in mice after repeated administration of Meth (*n* = 16, sensitizing dose, 2.5 mg/kg/day, s.c.) for 7 days (days 0 to 6, see timeline, Fig. 1A)^34^. Control noS animals received a daily saline injection (*n* = 22, s.c., × 7 days). Animals were first placed into locomotor chambers on days -1 and 7 for 1-hour acclimatization. Locomotor activity was recorded for an hour after Meth (1 mg/kg, S group) or saline injection (noS group, Fig. 1A). Analysis was conducted using TOTDIST, HACTV, MOVTIME, and STRCNT. As seen in Fig. 1B–E, Meth significantly increased locomotor and stereotype behaviors than saline control on days -1 and 7 (Fig. 1, S vs. noS). Meth-induced hyperactivity was further enhanced after repeated injection on day 7 (Fig. 1, *P* < .05, D7 vs. D-1 in the S mice, two-way ANOVA+ NK test), representing Meth sensitization. On the other hand, chronic saline treatment did not alter the locomotor activity (D-1 vs. D7 in noS). Detailed statistics were listed in Table 2.

**Table 2.** Significant Increase in Locomotor and Stereotype Response after 7-d Meth Administration.

	Locomotor behavior			
	Two-way ANOVA		Post hoc NK test	
	Saline vs. Meth		d-1 vs. d7 (saline)	d-1 vs. d7 (Meth)
	*P*	*F*_(1, 72)_=	*P*	*P*
TOTDIST	<0.001	41.706	0.599	<0.001
HACTV	<0.001	107.253	0.458	<0.001
MOVTIME	<0.001	101.932	0.218	<0.001
STRCNT	<0.001	115.599	0.756	<0.001

*P* and *F* values were determined by 2-Way ANOVA and NK tests.

**Figure 2.**
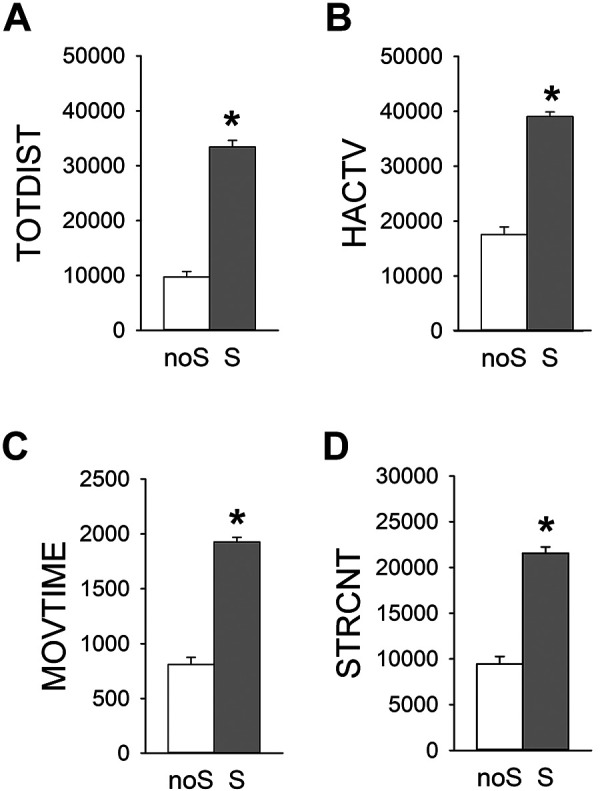
Relapse of Meth sensitization. Meth-induced behavior was examined at 2 weeks after the cessation of 7-day Meth (S) or saline (noS) treatment. Meth-induced (A–C) locomotor activity and (D) stereotype were significantly increased in the Meth -sensitized mice (or S mice) than non-sensitized mice (or noS mice). *P < 0.001, *t*-test.

Meth (1 mg/kg) -induced behavior was next examined at 2 weeks (Day 21) after 7d saline (*n* = 31, noS group) or Meth (*n* = 51, S group) treatment (Fig. 2). S mice maintained a significant increase in locomotor activity than noS mice (*P* < 0.001, *t*-test**;** Fig. 2). These data suggested that Meth-priming relapse was long-lasting.

### Partial Lesioning of Dopaminergic Terminals at NAc with 6-OHDA Reduced Meth Behavior Sensitization

**Figure 3.**
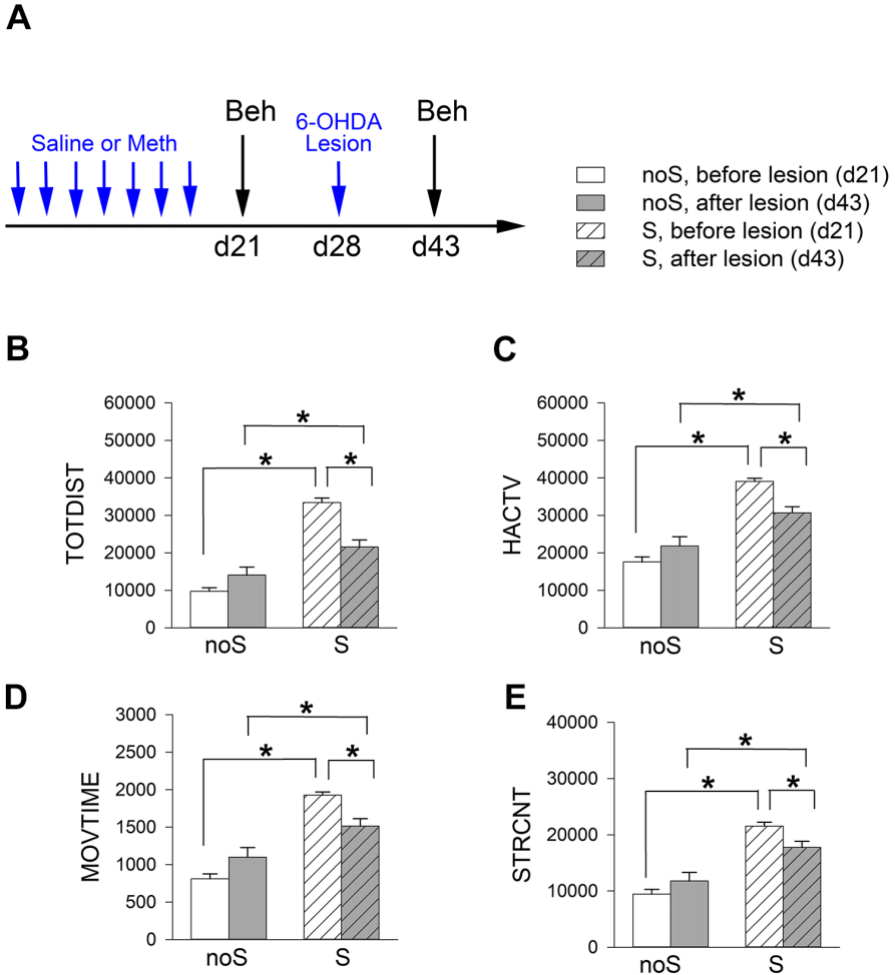
6-OHDA lesioning significantly attenuated Meth –induced hyperactivity and stereotype behaviors in Meth -sensitized mice. (A) Timeline of experiment. Mice received a daily dose of Meth (2.5 mg/kg) or saline from day 0 to 6, and bilateral NAc lesioning by 6-OHDA on day 28. Meth (1 mg/kg)-induced locomotor activity was recorded on day 21 and 43. Meth–induced hyperactivity (B–D) and stereotype behavior (E) were significantly attenuated by 6-OHDA. Lesioning of NAc did not reduce locomotor activity in the non-sensitized (noS) mice. Sensitized mice (S) received repeated Meth injections. Non-sensitized mice (noS) received a repeated saline injection. *P < 0.05, 2-way ANOVA + NK test.

The sensitized (*n* = 34) and non-sensitized (*n* = 18) mice were anesthetized. 6-OHDA (2.76 μg/µl × 1 μl) was stereotaxically administered into the bilateral NAc (Fig. 3A, timeline). Meth-induced locomotor behavior was examined at 2 weeks after lesioning. Meth–induced hyperactivity and stereotype behavior were significantly attenuated by 6-OHDA lesioning in the sensitized (S) mice (Fig. 3B–E, Table 3). In contrast, these responses in the non-sensitized (noS) mice were not affected by 6-OHDA (Fig. 3B–E, Table 3). These data suggest that Meth sensitization behavior is attenuated by lesioning of dopaminergic terminals in the NAc.

**Table 3.** Significant Reduction of Meth-Induced Hyperactivity and Stereotype Behaviors in Meth -Sensitized (S) Mice after 6-OHDA Lesioning.

Locomotor behavior	noS	S
	Lesioned vs. Non-lesioned	Lesioned vs Non-lesioned
	*p	*P*
TOTDIST	0.096	<0.001
HACTV	0.073	<0.001
MOVTIME	0.024	<0.001
STRCNT	0.15	0.002

*2-way ANOVA + NK test.

### TH Expression in the NAc

**Figure 4.**
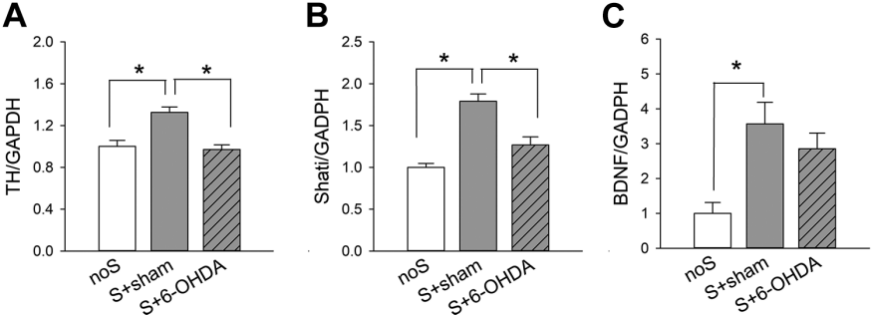
Expression of TH, Shati, and BDNF in the NAc after 6-OHDA lesioning. Meth-sensitized (S) mice were bilaterally injected with 6-OHDA or saline (sham) at NAc regions. On day 21 after injection, NAc tissues were collected for qRT-PCR to quantify the mRNA levels of TH, Shati, and BDNF, which were normalized to the reference gene GAPDH. Repeated Meth administration significantly upregulated the expression of (A) TH, (B) Shati, and (C) BDNF in the NAc (*P* < 0.05, noS vs. S+sham). These responses were significantly attenuated by local administration of 6-OHDA. noS: non-sensitized control; S+sham: sensitized mice receiving bilateral saline; S+6-OHDA: sensitized mice receiving bilateral 6-OHDA. *P < 0.05 (one-way ANOVA+NK test).

A total of 38 mice were used for this analysis. Of these, 18 were Meth-sensitized mice receiving bilateral 6-OHDA lesioning S+6-OHDA, 8 were Meth-sensitized mice receiving bilateral saline injection (S+sham), and 12 were non-sensitized (noS) mice. NAc tissues were collected for qRT-PCR analysis 21 days after lesioning or sham surgery. TH mRNA was significantly upregulated by repeated Meth administration (*P* < 0.05, S+sham vs. noS, 1-Way ANOVA+NK test, Fig. 4A). Local administration of 6-OHDA significantly attenuated TH expression (26.7% reduction) in the S mice (S+6-OHDA vs. S+sham, *P* < 0.05), suggesting a partial lesioning of DA innervation by 6-OHDA. No significant difference was found between S+6-OHDA and noS animals (*P* > 0.05).

### TH Expression in the Dorsal Striatum

The expression of TH in the dorsal striatum was next examined in 19 mice (*n* = 10, S+6-OHDA; *n* = 9, S+sham). Partial lesioning of NAc with 6-OHDA did not significantly alter the expression TH in the dorsal striatum (*P* = 0.584, *t*-test).

### Repeated Meth Administration Upregulated Shati and BDNF Expression in the NAc. 6-OHDA Lesioning Normalized the Expression of Shati

Previous studies have demonstrated that Meth addiction is associated with an increase in the expression of Shati and BDNF in NAc. Similar to these findings, we found that repeated Meth application significantly upregulated Shati and BDNF in NAc (Fig. 4B, C, noS vs. S+sham). 6-OHDA lesioning significantly mitigated Shati expression in the Meth-sensitized mice (S+6-OHDA vs. S+sham, Fig. 4B). There is a trend toward significance that 6-OHDA lesioning reduced BDNF expression in the S mice (Fig. 4C).

## Discussion

Repeated exposure to Meth alters the number of dendrites and the density of dendritic spines of dopaminergic neurons in the NAc^24^. The persistent changes of dopaminergic innervation result in increasing extracellular DA levels in the NAc and striatum^16,17^, altering DA and DOPAC/DA turnover in NAc^34,35^, and progressively augmenting behavioral responses to Meth and relapse. There is no effective pharmacological therapy to alleviate these physiological changes after repeated Meth exposure. In this study, dopaminergic terminals in NAc were partially lesioned by 6-OHDA. We demonstrated that Meth behavioral sensitization was significantly antagonized after the lesioning. Administration of significantly mitigated the upregulation of TH and Shati in Meth sensitized mice. The main finding of this study is that local administration of 6-OHDA reduced Meth sensitization in chronic Meth-treated animals. Our study is the first to examine selective lesioning of dopaminergic terminals in NAc to treat Meth addiction and supports a new surgical treatment strategy for Meth abuse.

In this study, we first characterized Meth behavioral sensitization. We found that Meth-induced hyperactivity was enhanced and lasted for 2 weeks after a 7d repeated Meth administration, representing Meth sensitization. The sustained hyperactive response has also been reported from >7 days^11,12^ to several months^13^ after repeated Meth administration. The mesolimbic dopaminergic pathway plays a significant role in the development of Meth behavioral sensitization^14,15^. Meth sensitization is associated with increases in extracellular dopamine (DA) levels, DOPAC/DA turnover, and upregulation of BDNF in the NAc, as we and others previously described^16–18,34^.

Limited studies have indicated that electrolytic or thermo- lesioning NAc reduced opioid dependence and relapse in animals^32^ and patients^36,37^. However, there are still multiple ethic concerns to apply these procedures for clinical use. For example, there is a lack of extensive preclinical studies to support the effectiveness and safety of these surgical procedures. NAc is the key node of the brain reward circuitry, electrolytic lesioning the NAc can non-selectively destroy the neurons or fibers passing through NAc, which may alter the processing of rewards-related stimuli (i.e., drug abuse, food, and sexual motivation).

The therapeutic effect of NAc lesioning in NAc for Meth addiction has not been previously investigated. To generate selective lesion of dopaminergic terminals in NAc, dopaminergic neurotoxin 6-OHDA was stereotaxically applied to the NAc in Meth-sensitized mice. We found that local administration of 6-OHDA reduced TH expression by 26.7% in the NAc of sensitized mice. More importantly, partially lesioning the NAc significantly reduced Meth sensitization behavior without affecting body weight (data not shown), suggesting that the low dose 6-OHDA may not compromise food reward. The physiological interaction of 6-OHDA lesioning and other rewarding stimuli warrants further investigation.

Shati (or Shati/Nat8 L) is a protein in the N-acetyltransferase family. Repeated Meth treatment increased Shati mRNA expression in the NAc^38^. The upregulation of Shati by repeated Meth is antagonized by dopamine D1 antagonist SCH23390^38^, suggesting that activation of dopaminergic transmission is required for Shati expression. Shati metabolizes aspartate to N-acetyl aspartate, which is further converted to n-acetyl aspartyl-glutamate, an agonist of metabotropic glutamate receptor type 3^39^. Overexpression of Shati in NAc attenuated Meth-induced locomotor sensitization and conditioned place preference in mice^19^. In contrast, blockage of Shati mRNA expression potentiated the Meth-induced increase of dopamine overflow in the NAc^20^. These data suggest that Shati is a Meth-sensitizing gene. The upregulation of Shati by Meth represents a homeostatic response of dopaminergic neurons in the NAc to inhibit the behavior action of Meth^38^. We found that the expression of Shati is upregulated after repeated Meth administration. 6-OHDA significantly downregulated Shati.

In conclusion, these data suggest that local administration of 6-OHDA mitigated Meth sensitization in chronic Meth-treated animals. Our data support a new surgical treatment strategy for Meth abuse.

## List of Abbreviation

6-OHDA: 6-hydroxydopamine
ATS: Amphetamine-type stimulants
BDNF: brain derived neurotrophic factor
DA: dopamine
HACTV: horizontal activity
Meth: methamphetamine
MOVTIME: movement time
NAc: nucleus accumbens
noS: non-sensitized mice
STRCNT: stereotypic movement number
TOTDIST: total distance traveled
qRT-PCR: quantitative Reverse Transcription PCR
S: sensitized mice
